# Electrochemical detection of white spot syndrome virus with a silicone rubber disposable electrode composed of graphene quantum dots and gold nanoparticle-embedded polyaniline nanowires

**DOI:** 10.1186/s12951-020-00712-4

**Published:** 2020-10-27

**Authors:** Kenshin Takemura, Jun Satoh, Jirayu Boonyakida, Sungjo Park, Ankan Dutta Chowdhury, Enoch Y. Park

**Affiliations:** 1 Laboratory of Biotechnology, Department of Bioscience, Graduate School of Science and Technology, Shizuoka University, 836 Ohya, Suruga-ku, Shizuoka, 422-8529 Japan; 2 Division of Pathology, Department of Aquaculture Research, Fisheries Technology Institute of Japan Fisheries Research and Education Agency, National Research and Development Agency, Tamaki Field Station, 224-1 Hiruta, Tamaki, Watarai, Mie 519-0423 Japan; 3 Division of Cardiovascular Diseases, Mayo Clinic College of Medicine and Science, Mayo Clinic, 200 First Street SW, Rochester, MN 55905 USA; 4 Laboratory of Biotechnology, Research Institute of Green Science and Technology, Shizuoka University, 836 Ohya, Suruga-ku, Shizuoka, 422-8529 Japan

**Keywords:** White spot syndrome virus, Electrochemical virus detection, Disposable electrode, Polyaniline, Gold nanoparticle, Graphene quantum dots

## Abstract

**Background:**

With the enormous increment of globalization and global warming, it is expected that the number of newly evolved infectious diseases will continue to increase. To prevent damage due to these infections, the development of a diagnostic method for detecting a virus with high sensitivity in a short time is highly desired. In this study, we have developed a disposable electrode with high-sensitivity and accuracy to evaluate its performances for several target viruses.

**Results:**

Conductive silicon rubber (CSR) was used to fabricate a disposable sensing matrix composed of nitrogen and sulfur-co-doped graphene quantum dots (N,S-GQDs) and a gold-polyaniline nanocomposite (AuNP-PAni). A specific anti-white spot syndrome virus (WSSV) antibody was conjugated to the surface of this nanocomposite, which was successfully applied for the detection of WSSV over a wide linear range of concentration from 1.45 × 10^2^ to 1.45 × 10^5^ DNA copies/ml, with a detection limit as low as 48.4 DNA copies/ml.

**Conclusion:**

The engineered sensor electrode can retain the detection activity up to 5 weeks, to confirm its long-term stability, required for disposable sensing applications. This is the first demonstration of the detection of WSSV by a nanofabricated sensing electrode with high sensitivity, selectivity, and stability, providing as a potential diagnostic tool to monitor WSSV in the aquaculture industry. 
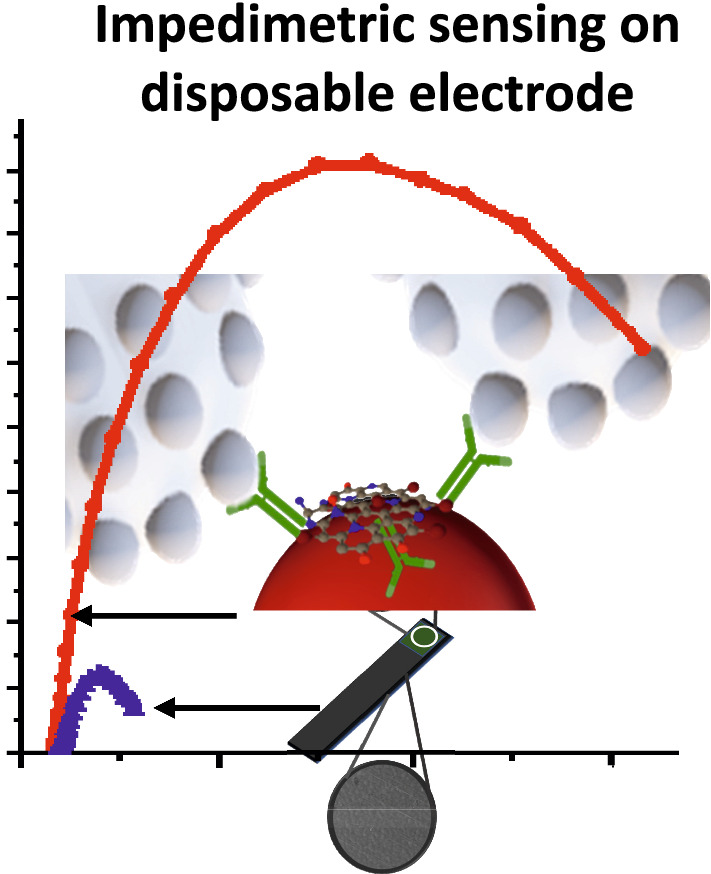

## Background

White spot syndrome virus (WSSV) infects shrimp and causes white spot disease (WSD), which is considered one of the most lethal virus pathogens in cultured shrimp [1]. WSSV was initially discovered in Taiwan and spread quickly to the entire world [2, 3]. The infection reaches a cumulative mortality of up to 100% within 10 d, causing rapid economic damage in fishery industries [4]. Recently, India reported an approximately several million US$ loss per year due to WSD [5]. WSSV spreads by vertical infection or cannibalism between shrimp and other invertebrate aquatic organisms, such as crab and crayfish [6], with a high mortality rate; a promising rapid detection method needs to be developed to prevent the onset of this epidemic.

Vaccination is always the most useful method for solving any viral infection. Using WSSV envelope proteins (e.g., VP19 and VP28), a few attempts to induce an immune response and protect shrimp from WSSV infection have already been reported [7–9]. However, the outcome of the vaccination method is still poor considering its practical use. To avoid the risk of WSD in shrimp farming, the only way is to quickly identify the infected shrimp and isolate it from the farm. Current methods for detecting WSSV rely on PCR techniques using viral DNAs or protein assays using a specific antibody [10, 11]. The limit of detection (LOD) is a few hundred DNA copies/ml within 4 to 12 h [12–14]. Likewise, dot blots, lateral flow assay, and enzyme-linked immunosorbent assay (ELISA) using antigen–antibody reactions have been generally used for the detection of WSSV envelope proteins [15–19]. The LOD is in the range of 1000 DNA copies/ml using the lateral flow assay and 120 ng/ml using ELISA. However, the availability of diagnostic PCR assays for use in aquaculture remains limited because they are costly and require highly skilled operators.

On the other hand, antigen detection methods are useful for rapid clinical diagnosis of viral infection [20] but fail to attain the desired sensitivity. In a recent study, a fluorescence resonance energy transfer (FRET)-based detection technique using graphene oxide detected WSSV with an LOD of 10 DNA copies/ml [21]. In another study, WSSV was detected with an LOD of 1.36 × 10^3^ DNA copies/µl using the electrochemical property of methylene blue conjugated to graphene oxide [22]. Although a few reports on WSSV detection with low sensitivity have been published, in terms of their stability and reliability, these sensors are not suitable for real-time applications.

Nanomaterials with unique physical, optical and electrochemical properties [23–28] have shown successful detection of viruses with high sensitivity [29–31]. Previously, we demonstrated hepatitis E virus detection with a fabricated biosensor electrode constituted by specific antibodies and nanomaterials based on an engineered impedimetric process [32]. In this report, we have developed a conducting sensor matrix fabricated with polyaniline, nitrogen and sulfur-codoped graphene quantum dots (N,S-GQDs) and gold nanoparticles (AuNPs) for the detection of WSSV. In an interfacial reaction, AuNPs embedded in polyaniline nanowires (AuNP-PAni) were synthesized and bound to N,S-GQDs via the Au–S affinity because the interaction between PAni and AuNPs provides excellent conductivity to the N,S-GQD@AuNP-PAni nanocomposite [33, 34]. The nanocomposite was deposited on a finely electropolymerized polyaniline-coated conductive silicon rubber (CSR) surface. The coating of the Ab-N,S-GQD@AuNP-PAni nanocomposite on the surface of the CSR significantly improved the conductivity of the CSR. On the other hand, the conductivity significantly decreased after capturing WSSV due to the increased charge transfer resistance (R_ct_) of the Ab-N,S-GQD@AuNP-PAni-coated electrode. This disposable electrode demonstrates the capability for detecting WSSV over a wide linear range with high specificity and sensitivity. The sensor stability was also tested over more than one month to confirm its applicability for on-site virus detection. Thus, our fabricated disposable electrode modified by a simple and uniform nanocomposite coating aiming for more convenient use allows accurate WSSV detection and is applicable to sensing WSSV in the aquaculture industry.

## Materials and methods

### Materials

PBS buffer, polyoxyethylene (20) sorbitan monolaurate (Tween 20), sodium acetate, hydrogen peroxide, sulfuric acid, methanol, potassium hydroxide (KOH), chloroform and acetone were purchased from Wako Pure Chemical Ind. Ltd. (Osaka, Japan). HAuCl_4_, N-(3-dimethylaminopropyl)-N′-ethylcarbodiimide hydrochloride (EDC), N-hydroxysuccinimide (NHS), and bovine serum albumin (BSA) were purchased from Sigma Aldrich Co., LLC (Saint Louis, MO, USA). Oleic acid was purchased from Nacalai Tesque Inc. (Kyoto, Japan). Tetramethylbenzidine (TMBZ) was purchased from Dojindo Laboratories (Kumamoto, Japan). Anti-WSSV VP28 antibody [ab26935] and a mouse monoclonal antibody [B219M] were purchased from Abcam Inc. (Cambridge, UK). Anti-HA antibody (New Caledonia/20/99) (H1N1) was purchased from Prospec-Tany Techno Gene Ltd. (Rehovot, Israel). Goat anti-rabbit IgG-HRP was purchased from Santa Cruz Biotechnology (CA, USA). Geno type 3 hepatitis E virus (HEV), rabbit anti-G3 HEV IgG antibody, and norovirus-like particle (NoV-LP) were provided by Dr Tian-Cheng Li (National Institute of Infectious Diseases, Japan). Zika virus (ZIKV) strain PRVABC-59 was provided by Professor Kouichi Morita (Institute of Tropical Medicine, Nagasaki University, Japan).

### Equipment

UV/vis absorption and fluorescence emission measurements were carried out using a filter-based multimode microplate reader (Infinite M200; TECAN, Ltd, Männedorf, Switzerland). Transmission electron microscopy (TEM) images were obtained with a TEM system (JEM-2100F; JEOL, Ltd., Tokyo, Japan) operated at 100 kV. Scanning electron microscopy (SEM) images were obtained with SEM equipment (JSM-6510LV; JEOL, Tokyo, Japan). A General Laboratory Homogenizer (OMNI International, USA) was used for WSSV sample preparation from shrimp. Western blotting was carried out using a Trans-Blot-SD (Bio-Rad, Japan) and transferred by using Immobilon ECL Ultra Western HRP Substrate (Merck, Japan). The membrane after western blotting was filmed by a VersaDoc 4000 MP (Bio-Rad, Japan). The centrifugation for virus sample collection proceeded using a Micro-cooled Centrifuge 3700 (KUBOTA, Japan). Powder X-ray diffraction (PXRD) analysis was carried out using a RINT ULTIMA XRD (Rigaku Co., Tokyo, Japan) with a Ni filter and a Cu-Kα source. Data were collected over 2θ = 5°–60° at a scan rate of 0.01°/step and 10s/point. Fourier transform infrared spectroscopy (FTIR) was recorded on FT/IR-6300 (JASCO, Japan). Zeta potential and dynamic light scattering (DLS) measurements were performed using a Zetasizer Nano series (Malvern Inst. Ltd., Malvern, UK). Conjugation of the Ab to the QDs and GNPs was confirmed with a plate reader (Bio-Rad, model 680, Hercules, USA). A high-resolution transmission electron microscopy (HRTEM) image was taken by a JEM-2100F at 200 kV (JEOL, Tokyo, Japan). Atomic force microscopy (AFM) analysis was achieved with a Nanoscope IV Pico Force Multimode atomic force microscope (Bruker, Santa Barbara, CA, USA) in contact mode [35]. Electrochemical cyclic voltammetry (CV) and electrochemical impedance spectroscopy (EIS) were carried out using an SP-150 (BioLogic Inc, Tokyo, Japan) in a conventional three-electrode cell consisting of platinum wire. Saturated Ag/AgCl was used as an electrolyzer (EC frontier, Tokyo, Japan).

### Synthesis of the AuNP-PAni nanocomposite

AuNP-PAni was synthesized using the interfacial polymerization method [34]. A 0.5 M aniline monomer in toluene mixture was prepared as an organic phase, and 3 mM HAuCl_4_ in 0.1 M HCl solution was slowly poured in as an aqueous phase to initiate the interfacial polymerization process. Polyaniline nanowires were gradually formed by oxidation of aniline into an aqueous phase, and the solution color became dark green within several minutes. At the same time, HAuCl_4_ was reduced to AuNPs and embedded within the polyaniline nanowires. The synthesized solution was centrifuged (room temperature, 5500×*g*) and redispersed using ultrapure water for purification. This purification process was repeated 3 times.

### N,S-GQD preparation and conjugation of anti-WSSV VP28 antibody

N,S-GQDs were synthesized using a hydrothermal system [36]. N,S-GQDs were quickly bonded with anti-WSSV VP28 antibody (Ab) using EDC/NHS covalent chemistry [37]. In brief, 0.1 M EDC was mixed with a solution containing Ab 5.1 µg, and EDC reacted with the carboxyl group of the Ab to create an active-ester intermediate within 30 min of stirring at 7 °C. To generate amine reactivity of the amino group with the surface of the GQDs, 0.1 M NHS and 1 ml of N,S-GQDs were added and continuously stirred at 7 °C over 16 h. The reaction solution was dialyzed using a 1 kDa dialysis bag to remove unreacted EDC and NHS. Finally, the solution of Ab-conjugated N,S-GQDs (Ab-N,S-GQDs) was preserved in 0.1 M PBS (pH 7.4) at 4 °C until use.

The conjugation of Abs to N,S-GQDs was confirmed using ELISA. Ab-conjugated N,S-GQDs were added to a polystyrene 96-well plate (100 µl) and incubated overnight at 4 °C. As a negative control, 100 µl of BSA was added to a separate well, and 100 μl of 5% skim milk solution was added and applied as a blocking agent after washing 3 times with PBST (containing 1 ml of Tween in 999 ml of PBS buffer). After blocking, the 5% skim milk was removed by washing 3 times with PBST. Anti-rabbit IgG-horseradish peroxidase was diluted to 1:4000 with 2% BSA, and 100 µl of this solution was added to the well and incubated at ambient temperature for 1 h. TMB (100 µl), a chromogenic substrate, was added to the well as a coloring reagent, and the solution appeared blue due to the reaction. The reaction was then stopped by adding 50 µl of 10% H_2_SO_4_, which changed the color of the solution from blue to yellow. The absorbance of the solution was measured using a microplate reader at 450 nm with a reference filter of 655 nm.

### Fabrication of the disposable electrode

Nanocomposite deposition on the sensor electrode produced high conductivity to the electrodes. Ab-N,S-GQD solution was mixed with AuNP-PAni solution and stirring for 16 h at 7 °C, where the Sulphur molecules, doped on N,S-GQD formed strong Au–S bonds with AuNPs via soft acid-soft base interaction.

In ultrapure water, 0.5 M sulfuric acid and 0.1 M aniline monomer were mixed for electrochemical deposition of polyaniline on conductive silicone rubber (CSR) by cyclic voltammetry (CV) in a three-electrode system. The CV curve was recorded at a scan rate of 20 mV/s in a potential range of 0–1 V for 15 cycles. The backside of the CSR electrode was covered by nonconducting tape to protect the polyaniline coating on the other side. Then, 15 µl of Ab-N,S-GQD@AuNP-PAni solution was drop-cast on the polymerized CSR/PAni. The formation of the Ab-N,S-GQD@AuNP-PAni nanocomposite was characterized by TEM and XRD.

### WSSV collection and pretreatment

A WSSV suspension was prepared according to a previously reported protocol [38]. In brief, the muscle tissue of moribund WSSV-infected shrimp was homogenized using GLH in 4× PBS volume, followed by centrifugation at 1000×*g* for 10 min at 4 °C. The supernatant was then filtered through a 0.22 µm cellulose acetate membrane. The filtrate containing WSSV was kept at − 80 °C before use in subsequent experiments.

### Detection of WSSV using the disposable electrode

The WSSV solution was diluted in series from 1.0 × 10^9^ DNA copies/ml to 10 DNA copies/ml using filtered 0.1 M PBS. Ten microliters of WSSV solution was dropped on the disposable electrode and incubated for 10 min at room temperature. WSSV bound the anti-WSSV VP28 Ab on the surface of the WSSV that was conjugated to Ab-N,S-GQD because of the specific binding between anti-WSSV VP28 Ab and the VP28 of WSSV. The electrode was mildly washed by dipping it in PBST to remove unbound viruses and then placed in an electrolytic solution. The charge resistance value (R_ct_) on the electrode was measured in the potential electrochemical impedance spectroscopy (PEIS) mode with a sinusoidal amplitude of 5 mV within a frequency range from 100 kHz to 0.1 Hz. The WSSV detection time using this disposable electrode was less than 15 min. For comparison, their DNA copy numbers were measured according to standard RT-PCR [39].

### Western blot analysis of WSSV-VP28

The resultant supernatants of WSSV were loaded on an 18% polyacrylamide gel under denatured conditions and transferred to a PVDF membrane using the Trans-Blot-SD system for 1 h at 15 mA. The membrane was then incubated in a blocking buffer (5% w/v skim milk in TBS containing 0.1% Tween 20 [TBS-T]) for 1 h at room temperature, followed by washing the membrane with TBS-T 3 times for 5 min. For protein detection, the membrane was incubated with a primary antibody, anti-WSSV-VP28 rabbit monoclonal antibody (1:2000), overnight at 4 °C and washed with TBS-T, followed by incubation with a secondary antibody (anti-IgG [Rabbit] pAb-HRP [1:10000]) for 1 h at room temperature. The immunoreactive bands were visualized and filmed for analysis.

### Selectivity and stability of the disposable electrode

IFV A (H1N1) and hepatitis E virus (HEV), various solution containing impurity, metal ions were used for the selectivity test of the Ab-N,S-GQD@AuNP-PAni/CSR electrode. In addition, solutions containing foreign substances was used to evaluate the practicability of the sensor in accordance with the cited references [40–42]. To confirm the stability, the disposable electrodes were preserved at 4 °C for 60 d, testing the performance of the electrodes every week.

## Results and discussion

### Characterization of Ab-N,S-GQD@AuNP-PAni on the CSR

**Scheme 1.**
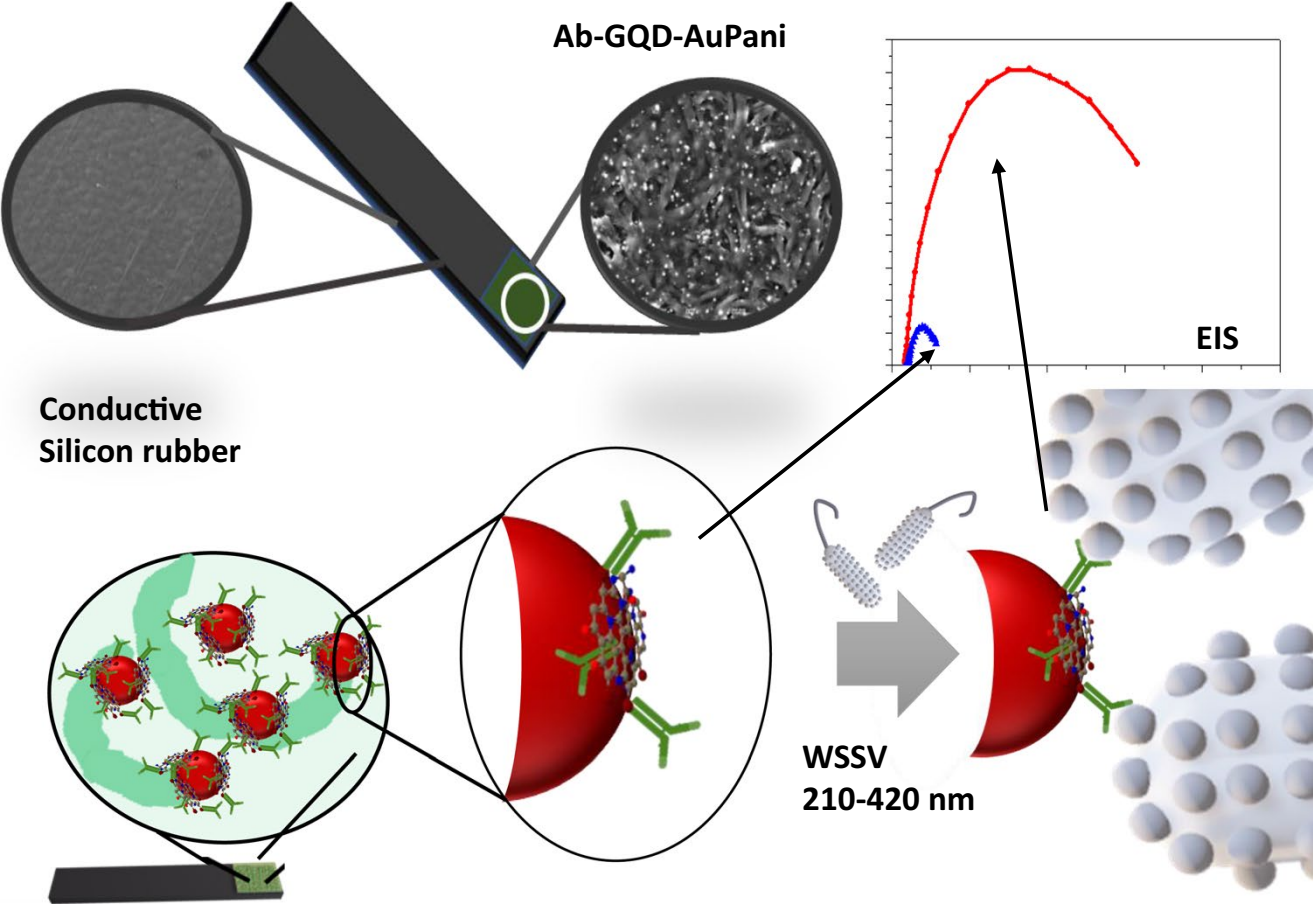
Overview of a highly conductive disposable electrochemical electrode for the detection of WSSV

**Fig. 1:**
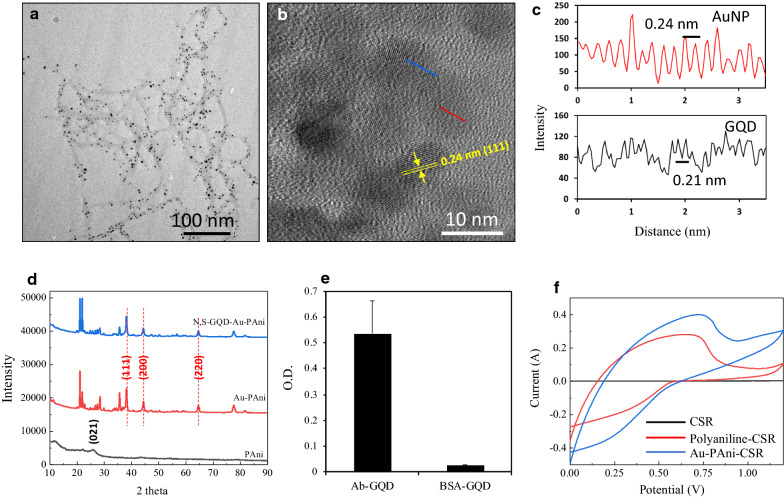
Characterization of Ab-N,S-GQD@AuNP-PAni. (**a**) TEM image of AuNP-PAni, (**b**) HR-TEM image of the N,S-GQD-AuNP nanocomposite, and (**c**) fringe analysis using ImageJ. (**d**) Powder XRD analysis of silicon, PAni/CSR, AuNP-PAni/CSR, and N,S-GQD-AuNP-PAni/CSR. (**e**) ELISA of Ab-N,S-GQDs. (**f**) Cyclic voltammetry diagrams of CSR, polyaniline-CSR and AuNP-PAni/CSR

The AuNP-PAni nanocomposite was deposited on a finely electropolymerized polyaniline-coated CSR electrode to form a AuNP-PAni-PAni/CSR electrode. The Ab-N,S-GQDs were then bound to the AuNP-PAni to prepare Ab-N,S-GQD@AuNP-PAni-PAni/CSR as shown in Scheme 1. In the N,S-GQD@AuNP-PAni nanocomposite, the AuNPs play an anchoring role between the N,S-GQDs and the polyaniline wires via soft acid—soft base interactions between Au and S. According to the TEM analysis, the diameter of the AuNP-PAni nanowires was 50–70 nm, where the AuNPs dispersed evenly in the polymeric chain, and their size ranged from 6–14 nm (Fig. 1a). The HRTEM image of the N,S-GQD@AuNP-PAni nanocomposite shows two distinct fringe patterns (Fig. 1b) of two crystalized structures of N,S-GQDs, and the AuNPs are deciphered as shown in Fig. 1c. The characteristic fringe of 0.24 nm for AuNPs is deciphered to the adjacent position of N,S-GQDs with a fringe distance of 0.21 nm, which is the distinctive fringe of the carbon lattice (Fig. 1c) [43, 44].

The structural properties of the N,S-GQD@AuNP-PAni nanocomposite were analyzed by XRD, as shown in Fig. 1d. PAni clearly revealed specific peak at 2θ = 26.0° corresponding to the (021). AuNP peaks are observed in the nanocomposite along with the characteristic peaks at 2θ = 23.6°, 25.5°, 28.2°, 38.2°, 44.3°, 64.4°, and 78.2° corresponding to the (100), (110), (111), (200), (220), and (221) planes, respectively (Fig. 1d) [45, 46]. After the N,S-GQDs were bound, the nanocomposite showed similar peaks and intensities, indicating that the attachment of GQDs does not induce any structural lattice changes of the AuNPs [47]. The graphitic layer shows a hump at 24° in the XRD spectrum, which is completely masked by the high-intensity peaks of AuNPs. To show the binding of N,S-GQD to AuNP-PAni, FT-IR analysis was performed. Similar peaks for PAni and AuNP-PAni were observed in addition to a characteristic peak at 2570 cm^–1^ for the thiol group of N,S-GQDs. A strong peak at 3300–3400 cm^–1^ was also observed for the amino or hydroxyl group as expected (Additional file 1: Fig. S1). These peaks indicate the attachment of N,S-GQDs on the AuNP-PAni nanocomposites. The conjugation of antibody was confirmed by ELISA as the absorbance value of the Ab-N,S-GQDs significantly increased compared with bare N,S-GQDs (Fig. 1e).

The electrochemical properties of the CSR electrode surface were measured by cyclic voltammetry. Despite the functional conducting matrix, the charge storage capacity of the bare CSR is very low, and the bare CSR shows a narrow curve, which significantly increases after the polyaniline coating (Fig. 1f). Additionally, a redox peak of polyaniline appears at + 0.8/ + 0.1 V, indicating the formation of the emeraldine salt of polyaniline [48]. After the formation of the nanocomposite, the conductivity of the disposable electrode shows an enhancement of the current density, indicating successful preparation of the sensor electrode for electrochemical analysis.

### Optimization of the sensing performance of Ab-N,S-GQD@AuNP-PAni

**Fig. 2:**
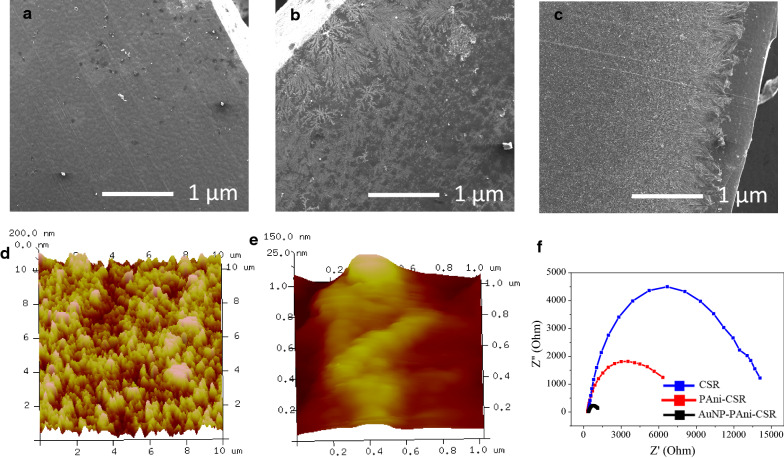
Surface appearances of CSR, PAni/CSR, and AuNP-PAni/CSR. **a**‒**c** SEM images and **d**‒**e** AFM images of CSR and AuNP-PAni/CSR, and **f** impedance Nyquist plot of CSR, PAni/CSR, and AuNP-PAni/CSR

The nanocomposite layer’s thickness on the CSR matrix is an essential parameter for maintaining the disposable electrode’s reproducibility. The thickness of the base matrix of polyaniline is directly proportional to the CV cycle number in the electropolymerization step. The resistance of the polyaniline-coated CSR becomes the lowest at 15 cycles because of the emeraldine salt formation of polyaniline (green). However, at 20 cycles or more, the redox reactions showed a decrease in conductivity due to predominantly overoxidized formation of pernigraniline of polyaniline (blue) (Additional file 1: Fig S2). Furthermore, after the electrolytic polymerization, SEM analysis of the CSRs showed that the polyaniline layer’s thickness became thicker with the increasing number of cycles (Additional file 1: Fig. S3A–C). A thick polyaniline layer can lead to a reverse effect on the conductivity of CSR. The 15 cycled PAni/CSR stability was tested over 50 cycles, showing excellent stability under the optimized polyaniline layer (Additional file 1: Fig. S4). The electrochemical properties of the CSRs did not change significantly after incubation in buffer for 24 h, confirming its stability as electrodes for sensor preparation (Additional file 1: Fig. S5). The thickness of the layer was further characterized by SEM and AFM. The bare CSR with a smooth surface becomes rough with coating of polymerized polyaniline (Fig. 2a‒b). The roughness of the polyaniline layer-coated CSR becomes relatively smoother again after drop-casting Ab-N,S-GQD@AuNP-PAni to microscale order (Fig. 2c). A similar observation was noted in the corresponding AFM images, as presented in Fig. 2d‒e. The CSR’s rough surface due to the bare polyaniline becomes relatively smooth after nanoconjugates formation, following the same trend as the SEM images. The AuNP-PAni forms a nanowire structure, and the binding of N,S-GQDs onto AuNPs makes the electrode smoother after the coating of nanocomposite to cover the porous structure of polyaniline. When the AuNP-PAni was modified on CSR, electrical conductivity significantly improved compared with AuNPs-modified CSR (Additional file 1: Fig. S6). In this study, the electric resistance was used as an indicator, and it was shown that AuNPs are a suitable material for this purpose because they lead to higher conductivity by forming a complex with polyaniline nanowires.

After optimizing the PAni electropolymerization and thickness of the sensor electrode, the changes in the electrochemical properties were investigated by EIS. The conductivity and dielectric properties of the CSR surface gradually decreased after polyaniline and successive Ab-N,S-GQD-AuNP-PAni conjugation (Fig. 2f), indicating successful formation of a sensor electrode suitable for virus detection.

Furthermore, the sensing area was optimized for virus detection. Electrodes with different sensor areas from 2 mm^2^ to 25 mm^2^ were prepared, and WSSV was detected by following the same procedure (Additional file 1: Fig. S7). The larger the sensor area was, the more remarkable the change in the Rct value. The sensing area’s size indicates the size of the contact surface with the virus during the antigen–antibody reaction. It suggests that the larger the area is, the more virus that binds to the sensor.

On the other hand, the sensor with a large area has a low correlation coefficient (R^2^ value) and a high error range, particularly in the high concentration range. As the area increases, it is difficult to obtain uniformity between electrodes with simple modification by only dropping nanomaterials, resulting in a low R^2^ value in virus detection. The electrode with a sensing area of 10 mm^2^, which gave the most reliable result, was used as the optimum detection electrode.

### Detection of WSSV

**Fig. 3:**
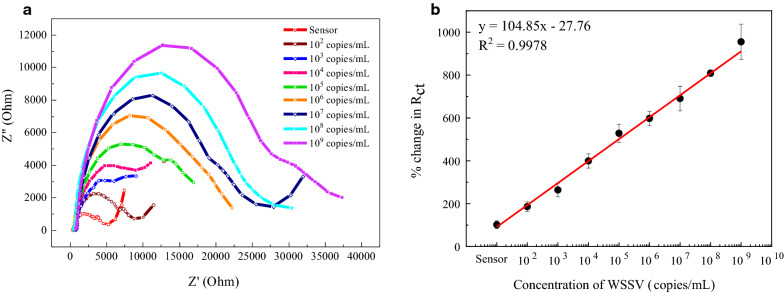
WSSV detection using the disposable electrode. **a** Nyquist plots for different concentrations of WSSV in the range of 10^2^–10^9^ DNA copies/ml. **b** Calibration curve of the corresponding impedance. Each detection was performed three times and data are given as average ± SD (n = 3)

The Nyquist impedance plots of the disposable electrode after incubation of different concentrations of the virus from 10^2^–10^9^ copies/ml are shown in Fig. 3A. The EIS responses of the sensor electrodes increase with the concentration of WSSV due to the high resistance accumulation between the virus-loaded nanocomposite and CSR. When WSSV binds to the sensing electrode, a large number of nonconducting virus particles cover the conducting surface of Ab-N,S-GQD@AuNP-PAni/CSR, increasing the charge transfer resistance (R_ct_). The percentage change of the signal difference between the R_ct_ values of the corresponding virus-loaded electrode and the bare electrode was adopted as the measurement signal. The calibration plot displays an excellent linear relationship between R_ct_ and the WSSV concentration (Fig. 3b). The LOD was found as low as 48.4 copies/ml, calculated by 3σ/S (S is the slope of the linear calibration plot, and σ is the unbiased standard deviation from the lowest signal of the detection result) [49]. This value is extremely low and sensitive enough to detect the real analyte [50]. After WSSV detection, the surface of the virus-loaded electrode exhibited a significantly increased roughness, indicating the presence of WSSV on the electrode (Additional file 1: Fig. S8).

We compared our sensing performance with various WSSV detection methods in Table 1. Many studies have successfully detected DNA as the target analyte. However, it is not easy to implement on-site and rapid detection because of the need to extract DNA from the WSSV. On the other hand, antigen detection with high sensitivity has not been reported earlier, except for electrochemical methods. Our detection system is useful because it shows high sensitivity, simplicity, and adaptability for on-site detection.

**Table 1:** Comparison of the sensing performance of our proposed sensor with various WSSV detection methods

Materials/Method of detection	Target virus (Analyte)	Detection range	LOD	Refs
Piezoelectric microcantilever sensors	WSSV (DNA)	50 to 10^5^ virions/ml	100 virions/ml	[51]
Lateral flow assay	WSSV (DNA)	36–1784 viral copies/ng	356 viral copies/ng	[52]
Surface plasmon resonance	WSSV (Antigen)	5 to 50 ng/ml	2.5 ng/ml	[53]
Loop-mediated isothermal amplification	WSSV (DNA)	0.05 to 1 μg/reaction (LAMP products)	2 × 10^2^ copies	[54]
Electrochemical	WSSV (Antigen)	1.37 × 10^−3^ to 1.37 × 10^7^ copies/μL	1.36 × 10^−3^ copies/μl	[22]
Enzyme-linked immunosorbent assay	WSSV (Antigen)	15–240 ng/well	250 pg/well	[55]
Polymerase chain reaction	WSSV (DNA)	9.0 × 10^1^–2.0 × 10^4^ copies/μg	4 copies/sample	[14]
Impedance electrochemical detection	WSSV (Antigen)	10^2^ to 10^9^ DNA copies/ml	48.4 DNA copies/ml	This work

### Selectivity and stability of the disposable electrode

**Fig. 4:**
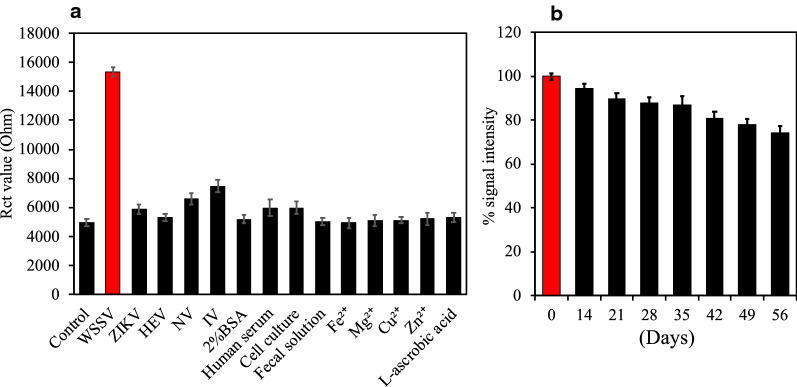
**a** Selectivity test of Ab-N,S-GQD@AuNP-PAni/CSR for WSSV detection compared with nontarget viruses. The concentration of IFV and HEV used was 10 pg/ml, while that of ZIKV and NoV was 10^4^ copies/ml. Human serum used 100% in solution. Fecal solution indicates the supernatant of centrifuged solution 1 g of feces in 1 ml of PBS buffer. Other ions and L-ascorbic acid were prepared to 1 mM. **b** Stability test of the disposable electrode. The electrode was stored in the refrigerator for 56 days, and the detection performance was investigated every week from the 2nd week. The Rct value on the first day was set to 100%

As the antibody directs the interaction between the analyte and the sensor electrode, the sensor should possess high selectivity. However, the specificity test of the sensor is still crucial for clarifying any possible cross-reactivity in real application. To confirm the specificity towards WSSV, various other viruses and some materials were tested to the sensor electrode. The sensor responses, except for WSSV (Fig. 4a), were similar to that of the bare electrode, indicating the sensor specificity for the target virus. The high selectivity of the sensors was achieved by a close coating of Ab-N,S-GQD-AuNP-PAni and effective cleaning with PBST. When many foreign substances were present in the real matrix, and the non-specific adsorption occurred on the sensor surface, the substances other than the target WSSV were removed with a highly efficient washing solution. This led to high selectivity of our proposed detection method.

The effect of interferences on sensor’s performance and recovery ratio of target analyte were also investigated [56, 57]. A fixed concentration of 10^4^ copies/ml WSSV was mixed with different matrixes and then similarly detected by the sensor electrode. The concentration of the recovered WSSV was calculated using a calibration curve (Fig. 3b) based on the obtained Rct values. The recovery ratio was compared, as shown in Table 2. When the WSSV was in PBS, l-ascorbic acid, Fe^2+^, and Cu^2+^ ions, the recovery ratio was almost 110%, while in case of Mg^2+^ and Zn^2+^, it was 90%. These results indicate that some ions affect sensing performance with a standard deviation of ± 13%. There was around 4% error in the recovery ratio, indicating that this system shows reasonable performances even in real matrix samples.

**Table 2:** Detection of WSSV in various interferences

Suspension^a^	Average Rct value^b^	Concentration by Rct (copies/ml)^c^	Recovery ratio (%)^d^	Relative error (%)^e^
PBS	15,833	10,915.0	109.2	± 2.4
L-ascorbic acid (1 mM)	15,244	10,987.2	109.9	± 3.8
Fe^2+^ (1 mM)	15,784	11,577.6	110.8	± 4.2
Mg^2+^ (1 mM)	15,484	9,187.9	91.9	± 3.8
Cu^2+^ (1 mM)	15,662	12,523.3	125.2	± 2.9
Zn^2+^ (1 mM)	15,422	8512.7	88.1	± 3.3

^a^WSSV concentration in suspension is 10^4^ copies/ml

^b^Average Rct value of WSSV detection (n = 3)

^c^WSSV concentration was calculated using the calibration curve (Fig. 3b)

^d^Recovery was defined as $\frac{\left\{\mathrm{Concentration by Rct}-{10}^{4}\right\}\mathrm{ copies}/\mathrm{mL}}{{10}^{4} \mathrm{copies}/\mathrm{mL}}\times 100$

^e^Recovery error was defined as $\sqrt {\frac{1}{{n - 1}}\sum\limits_{{k = 1}}^{n} {\left( {x_{i}  - \mathop x\limits^{ - } } \right)} ^{2} }$, where ${x}_{i}$ and $\stackrel{-}{x}$ denote Rct and average Rct values (n = 3), respectively

The stability of the disposable electrode was tested for 8 weeks to observe its applicability for long-term usage. As depicted in Fig. 4b, the signal intensity of Rct after loading of 10^4^ copies/ml virus remained at 86% until 35 days. However, it dropped to 73.4% after 56 days of storage due to degradation of the antibody.

To extend its application to other types of analytes, we prepared two different electrodes conjugated with different anti-HEV and anti-HA antibodies and detected their corresponding target viruses. These results demonstrate that the Nyquist impedance in both cases increases with increasing virus concentration (Additional file 1: Fig. S9A and B), and their corresponding calibration lines show excellent linearity (Additional file 1: Fig. S10A and B). The limit of detection was calculated as 34.6 DNA copies/ml for G3 HEV and 0.98 fg/ml for influenza virus A.

### Real virus analysis

**Fig. 5:**
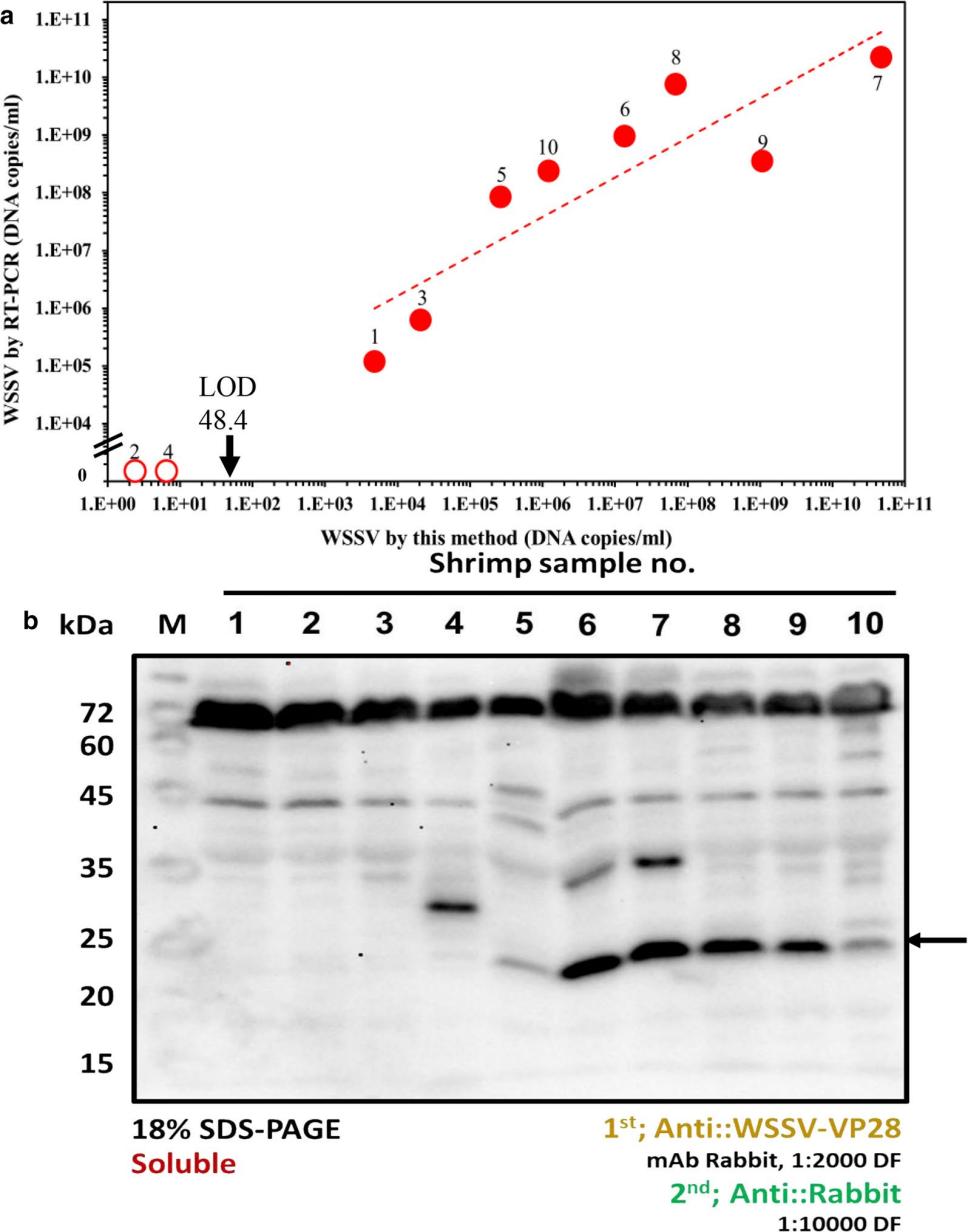
**a** Comparison of electrochemical detection and RT-PCR methods. The open circles (opened circle) indicate a negative and the red circles (closed circle) a positive result as judged by the RT-PCR result. The arrow indicates the limit of detection. **b** Western blot analysis of WSSV-VP28 from shrimp samples using anti-VP28 antibody as a primary antibody. The arrow indicates VP-28

After successful detection of WSSV in a buffer medium, real samples were collected from 10 WSSV-infected shrimp and tested with the sensor. Their DNA copy numbers were compared with the results obtained from this electrochemical detection technique. The detection results are summarized in Table 3 and Fig. 5a. According to the RT-PCR data, sample Nos. 2 and 4 do not contain any WSSV, showing 2.4 and 6.5 copies/ml according to our electrochemical method, and can be ignored. The electrochemical detection results for sample Nos. 8 and 9 significantly deviate from the RT-PCR results. However, the overall trend of the RT-PCR results for the samples shows excellent similarity to the trend of the electrochemical sensor results, confirming the reproducibility of the sensor. In the western blot analysis, the virus titer above 10^7^ copies/ml shows VP-28 protein bands at approximately 22 kDa, but less than 10^7^ copies/ml could not be detected (Fig. 5b). This indicates that our sensing system shows a 6–7 order of magnitude higher sensitivity than western blot. This method, which can detect WSSV from specimens in less than 20 min, is much faster than the time-consuming RT-PCR, which is currently used as a gold standard. Although the correlation coefficient between the two methods is 90%, the developed method can be used to judge WSSV infection in a short time with easy handling.

**Table 3:** Details of the detection results for real sample detection using the electrochemical method and RT-PCR

Sample no	R_ct_ value ± SD (n = 3)	WSSV concentration (DNA copies/ml)		VP28 detection	Shrimp
		by EIS^a^	by RT-PCR		
Control	2680 ± 146	0	–		
1	7179 ± 238	4.8 × 10^3^	1.2 × 10^5^	No	Live
2	2797 ± 72	2.4 × 10^0^	0	No	Live
3	8988 ± 108	2.0 × 10^4^	6.2 × 10^5^	No	Dead
4	4572 ± 143	6.5 × 10^0^	0	No	Live
5	12,101 ± 490	2.6 × 10^5^	8.4 × 10^7^	No	Dead
6	16,946 ± 406	1.4 × 10^7^	9.6 × 10^8^	Yes	Dead
7	26,949 ± 140	4.7 × 10^10^	2.2 × 10^10^	Yes	Dead
8	18,946 ± 893	6.9 × 10^7^	7.5 × 10^9^	Yes	Dead
9	22,308 ± 195	1.0 × 10^9^	3.5 × 10^8^	Yes	Dead
10	13,988 ± 406	1.2 × 10^6^	2.4 × 10^8^	Yes	Dead

^a^The copy number of WSSV was determined from the calibration line (Fig. 3C) using the measured R_ct_ value

## Conclusion

A disposable electrode consisting of an Ab-N,S-GQD@AuNP-PAni nanocomposite on a CSR electrode was fabricated in this work for the rapid and sensitive detection of WSSV within 20 min. This disposable sensor showed a low Rct value in the impedance spectrum as a bare sensor, which significantly increased with the target virus concentration over a wide linear range from 10^2^ to 10^9^ DNA copies/ml, with a LOD of 48.4 copies/ml. The proposed disposable electrode's applicability was successfully demonstrated, with high selectivity and long-term stability of 5 weeks. The sensing capability was also tested for other viruses, indicating its versatile applicability for future usage. The sensor was applied to detect the real WSSV from WSSV-infected shrimp in aquaculture and found to be comparable with RT-PCR analysis, which confirmed its applicability as an excellent monitoring system for real-time virus detection. This detection system will play an essential role in controlling the spread of WSSV for on-site detection systems at shrimp farms that do not have adequate testing facilities.

## Supplementary information

**Additional file 1: Figure S1** Analysis of functional groups on the surface of named materials by FT-IR infrared absorption spectra. **Figure S2.** Impedance analysis of the PAni-PAni/CSR sensor electrode after 5 to 20 cycles of electrodeposition. **Figure S3** (A–C) The difference in thickness of the polyaniline layer deposited on CSR was observed by SEM. **Figure S4.** Cyclic voltammetry analysis of the Ab-N,S-GQD@AuNP-PAni-PAni/CSR sensor electrode after 2 to 50 cycles. **Figure S5.** Cyclic voltammetry of CSR day1 (black line) and day2 (red line). **Figure S6.** Comparison of the Rct values for AuNP-coated CSR (blue line) and AuNP/PAni-coated CSR (orange line). **Figure S7.** Detection result of WSSV using different surface areas of Ab-N,S-GQD@AuNP-PAni-PAni/CSR. **Figure S8.** AFM images of the bare disposable sensor electrode (A) and WSSV-bound electrode (B). **Figure S9**. Nyquist impedance plots of the Ab-N,S-GQD@AuNP-PAni-PAni/CSR sensor electrode before and after loading A) HEV and B) influenza virus. **Figure S10.** Detection results of genotype 3 HEV (A) and influenza virus A (H1N1) (B) using the Ab-N,S-GQD@AuNP-PAni/CSR sensor electrode with their corresponding antibodies attached.

## Data Availability

All data generated or analyzed during this study are included in this manuscript and its additional information.
