# Supplementary material for: Electrochemical detection of white spot syndrome virus with a silicone rubber disposable electrode composed of graphene quantum dots and gold nanoparticle-embedded polyaniline nanowires
Source: J Nanobiotechnology. 2020 Oct 27;18:152. doi: 10.1186/s12951-020-00712-4 (PMC7590724; doi:10.1186/s12951-020-00712-4)
Supplement: Supplementary file 1 — Additional file 1: Figure S1 Analysis of functional groups on the surface of named materials by FT-IR infrared absorption spectra. Figure S2. Impedance analysis of the PAni-PAni/CSR sensor electrode after 5 to 20 cycles of electrodeposition. Figure S3 (A–C) The difference in thickness of the polyaniline layer deposited on CSR was observed by SEM. Figure S4. Cyclic voltammetry analysis of the Ab-N,S-GQD@AuNP-PAni-PAni/CSR sensor electrode after 2 to 50 cycles. Figure S5. Cyclic voltammetry of CSR day1 (black line) and day2 (red line). Figure S6. Comparison of the Rct values for AuNP-coated CSR (blue line) and AuNP/PAni-coated CSR (orange line). Figure S7. Detection result of WSSV using different surface areas of Ab-N,S-GQD@AuNP-PAni-PAni/CSR. Figure S8. AFM images of the bare disposable sensor electrode (A) and WSSV-bound electrode (B). Figure S9. Nyquist impedance plots of the Ab-N,S-GQD@AuNP-PAni-PAni/CSR sensor electrode before and after loading A) HEV and B) influenza virus. Figure S10. Detection results of genotype 3 HEV (A) and influenza virus A (H1N1) (B) using the Ab-N,S-GQD@AuNP-PAni/CSR sensor electrode with their corresponding antibodies attached. [file 12951_2020_712_MOESM1_ESM.docx]

**Supplementary Information**

**Electrochemical detection of white spot syndrome virus with a silicone rubber disposable electrode composed of graphene quantum dots and gold nanoparticle-embedded polyaniline nanowires**

Kenshin Takemura^1^, Jun Satoh^2^, Jirayu Boonyakida^1^, Sungjo Park^3^, Ankan Dutta Chowdhury^4^, Enoch Y. Park^1,4,^^[[1]](#footnote-1)^

^1^ *Laboratory of Biotechnology, Department of Bioscience, Graduate School of Science and Technology, Shizuoka University, 836 Ohya, Suruga-ku, Shizuoka 422-8529, Japan*

^2^*Division of Pathology, Department of Aquaculture Research, Fisheries Technology Institute of Japan Fisheries Research and Education Agency, National Research and Development Agency, Tamaki Field Station, 224-1 Hiruta, Tamaki, Watarai, Mie 519-0423, Japan*

^3^ *Division of Cardiovascular Diseases, Mayo Clinic College of Medicine and Science, Mayo Clinic, 200 First Street SW, Rochester, MN, 55905, USA*

^4^ *Laboratory of Biotechnology, Research Institute of Green Science and Technology, Shizuoka University, 836 Ohya, Suruga-ku, Shizuoka 422-8529, Japan*


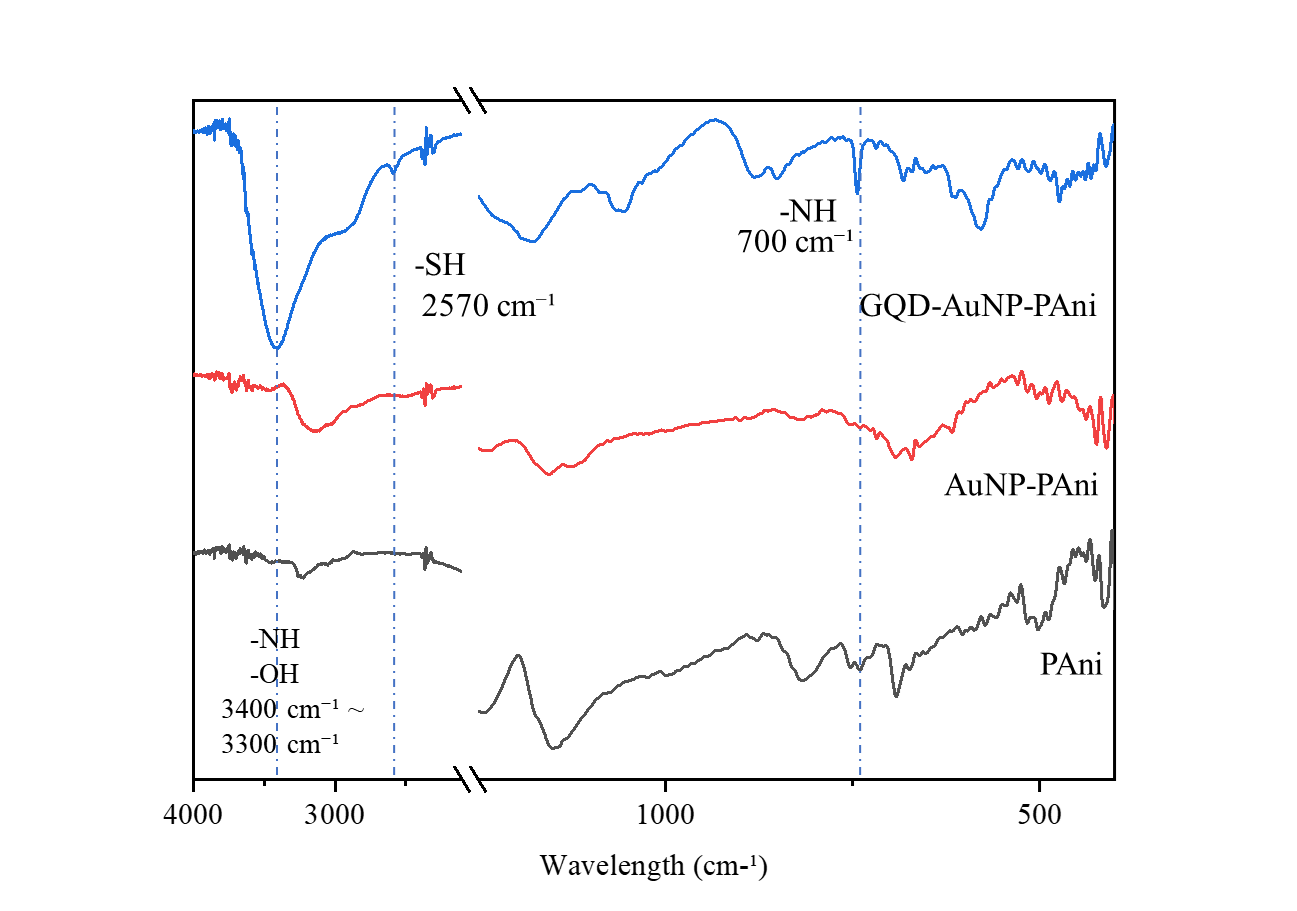


**Fig. S1** Analysis of functional groups on the surface of named materials by FT-IR infrared absorption spectra.

**Fig. S2** Impedance analysis of the PAni/PAni@CSR sensor electrode 5 to 20 cycles of electrodeposition.


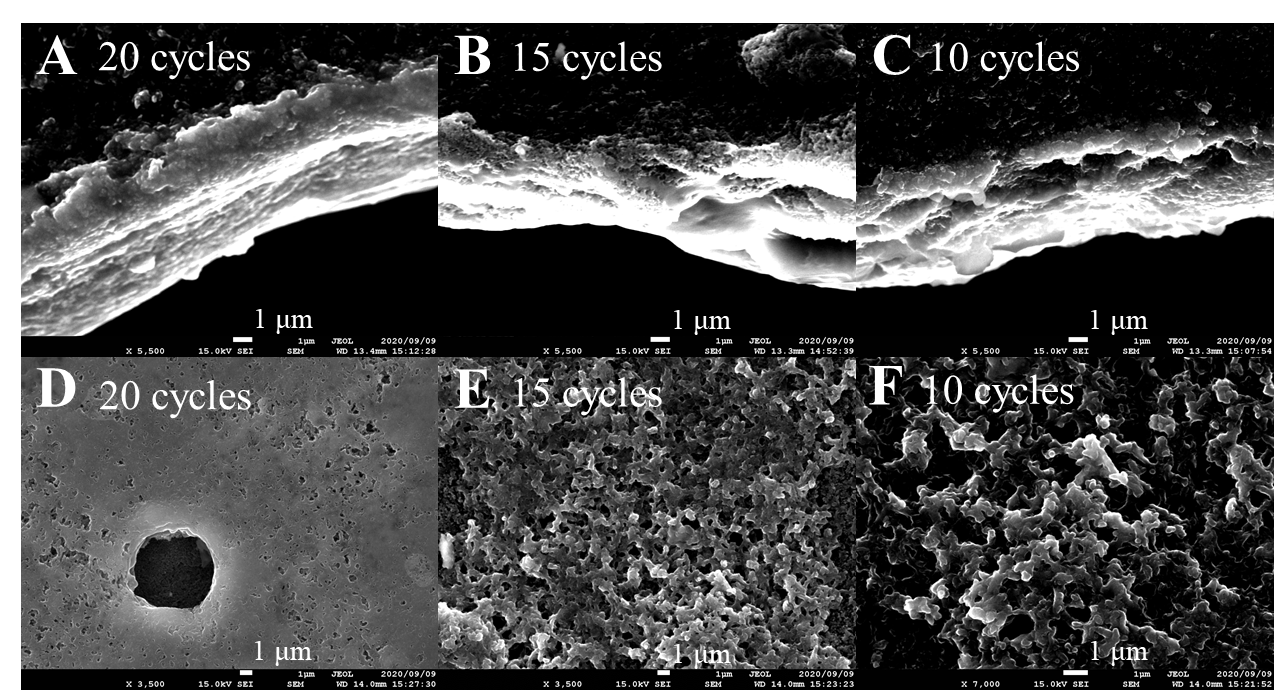


**Fig. S3** (**A–C**) SEM observed the difference in thickness of the polyaniline layer deposited on CSR.


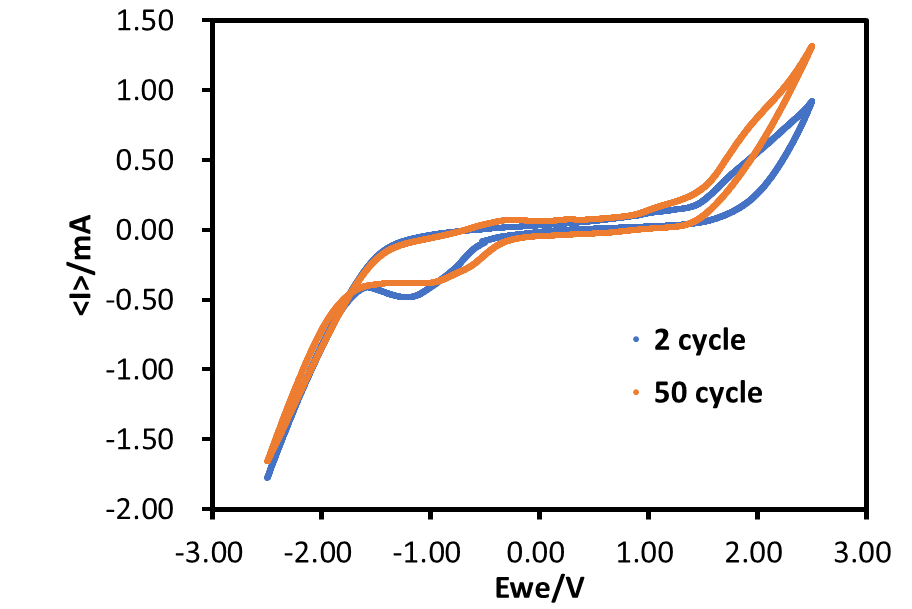


**Fig. S****4** Cyclic voltammetry analysis of the Ab-N,S-GQDs@AuNP-PAni/PAni@CSR sensor electrode 2 to 50 cycles.


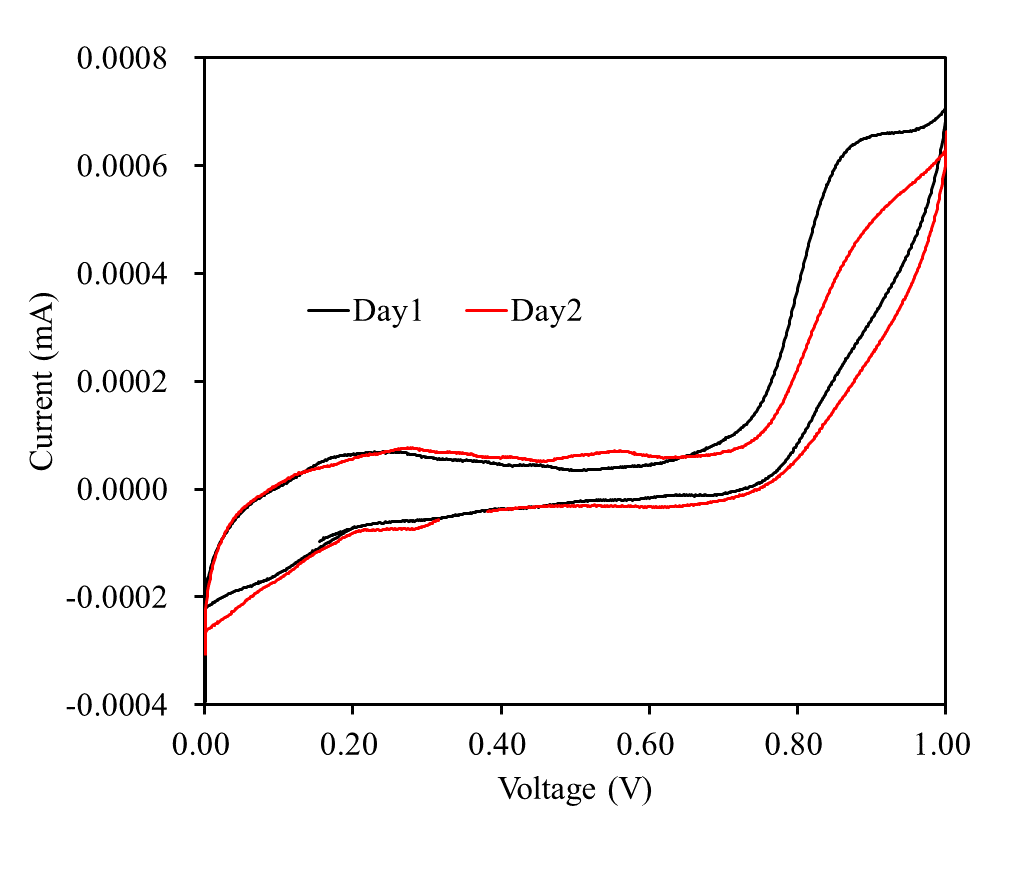


**Fig. S5**. Cyclic voltammetry of CSR at day1 (black line) and day2 (red line).


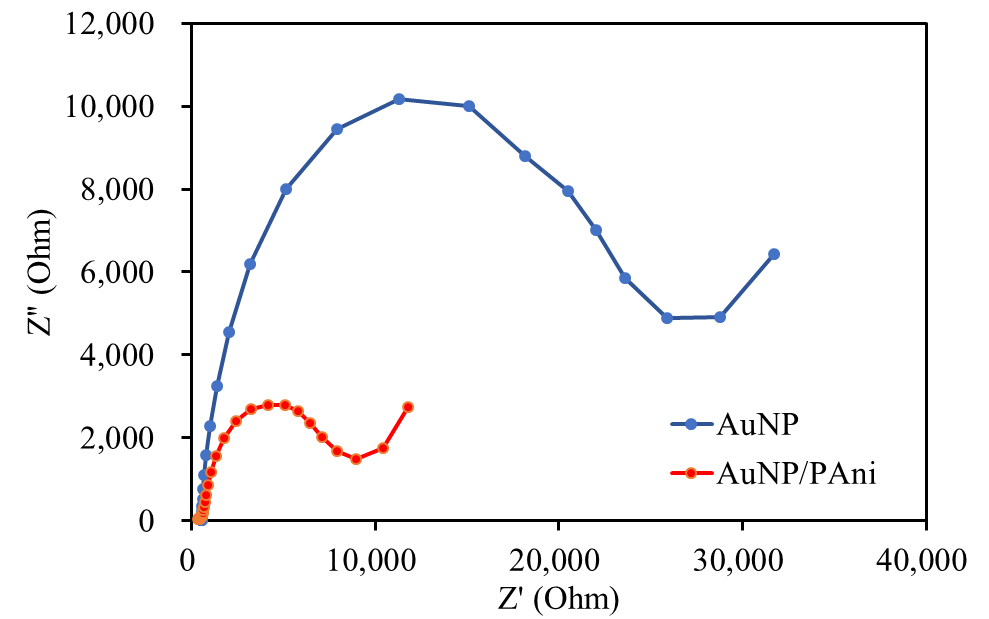


**Fig. S6** Comparison of Rct value with AuNP-modified CSR (blue line) and AuNP/PAni-modified CSR (orange line)


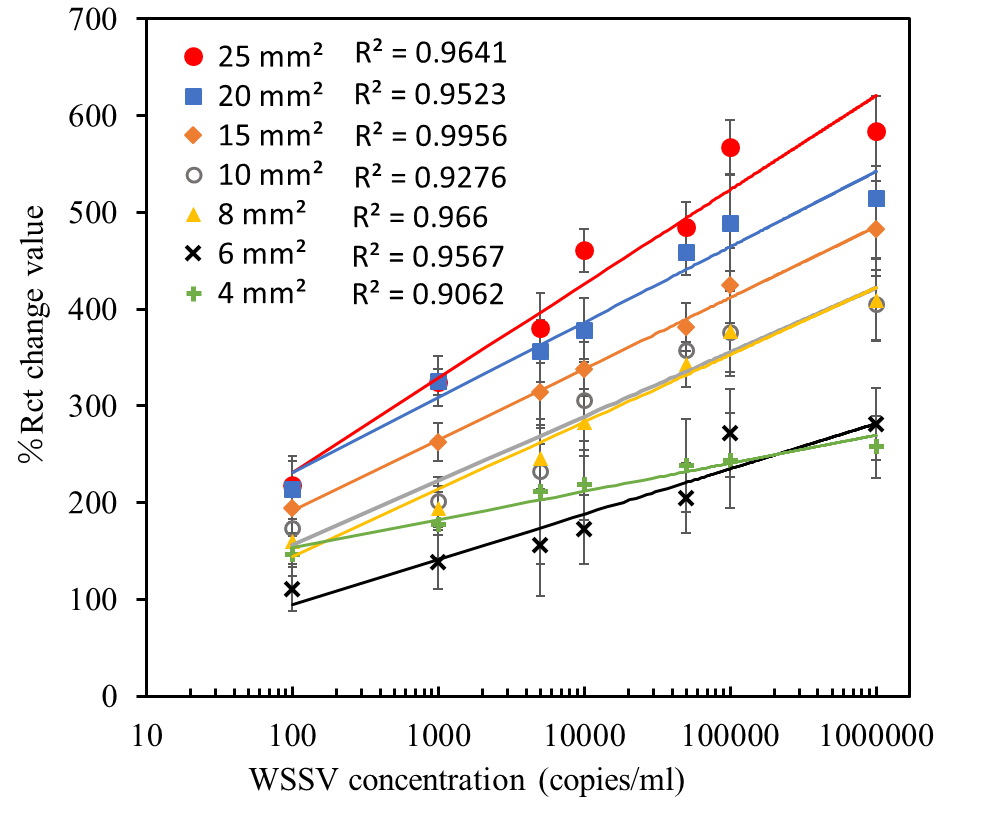


**Fig. S****7** Detection result of WSSV using different surface area of Ab-GQD@AuPAni/PAni@CSR.

**Fig. S8** AFM images of the bare disposable sensor electrode (A) and with WSSV bound electrode (B).


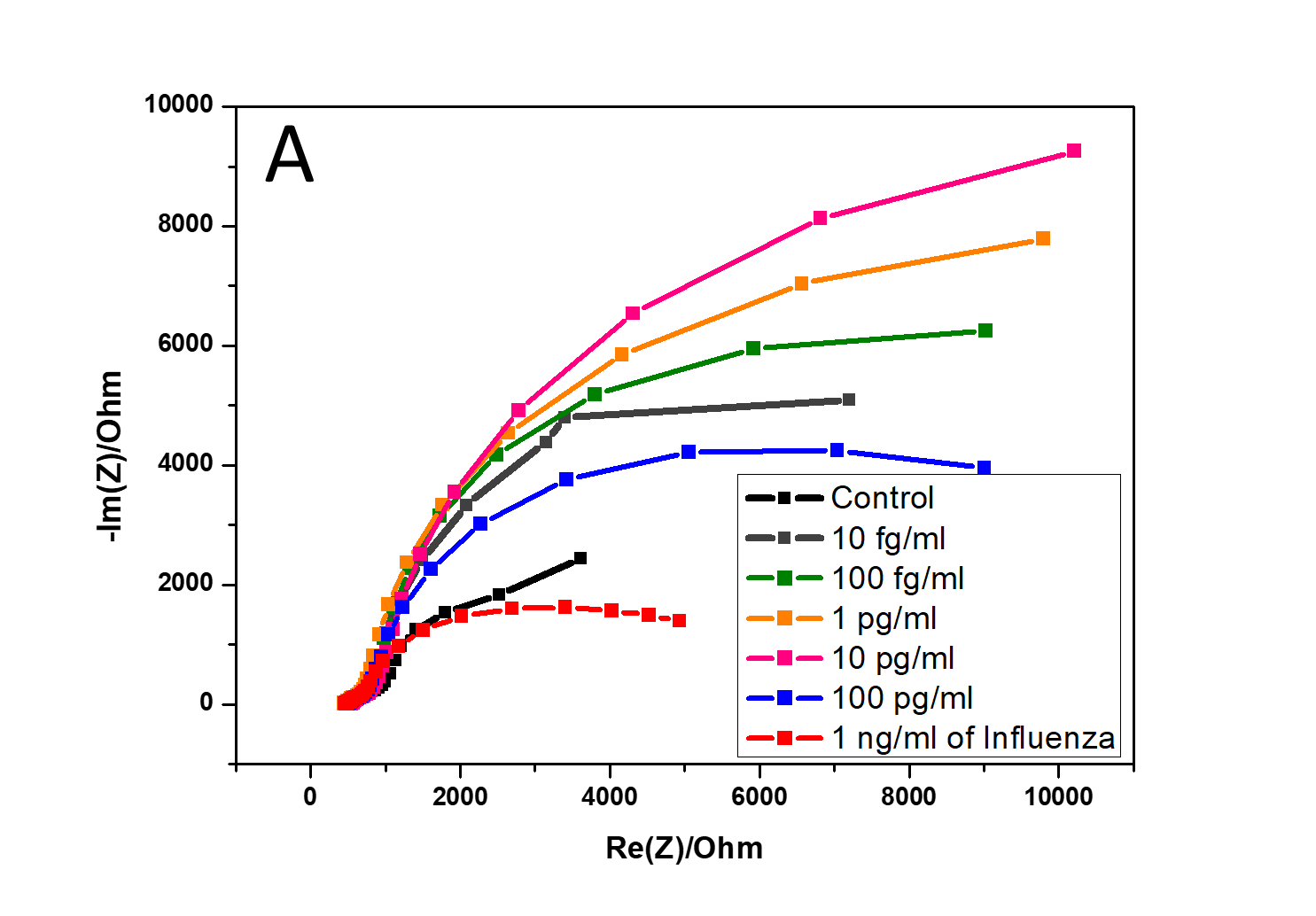


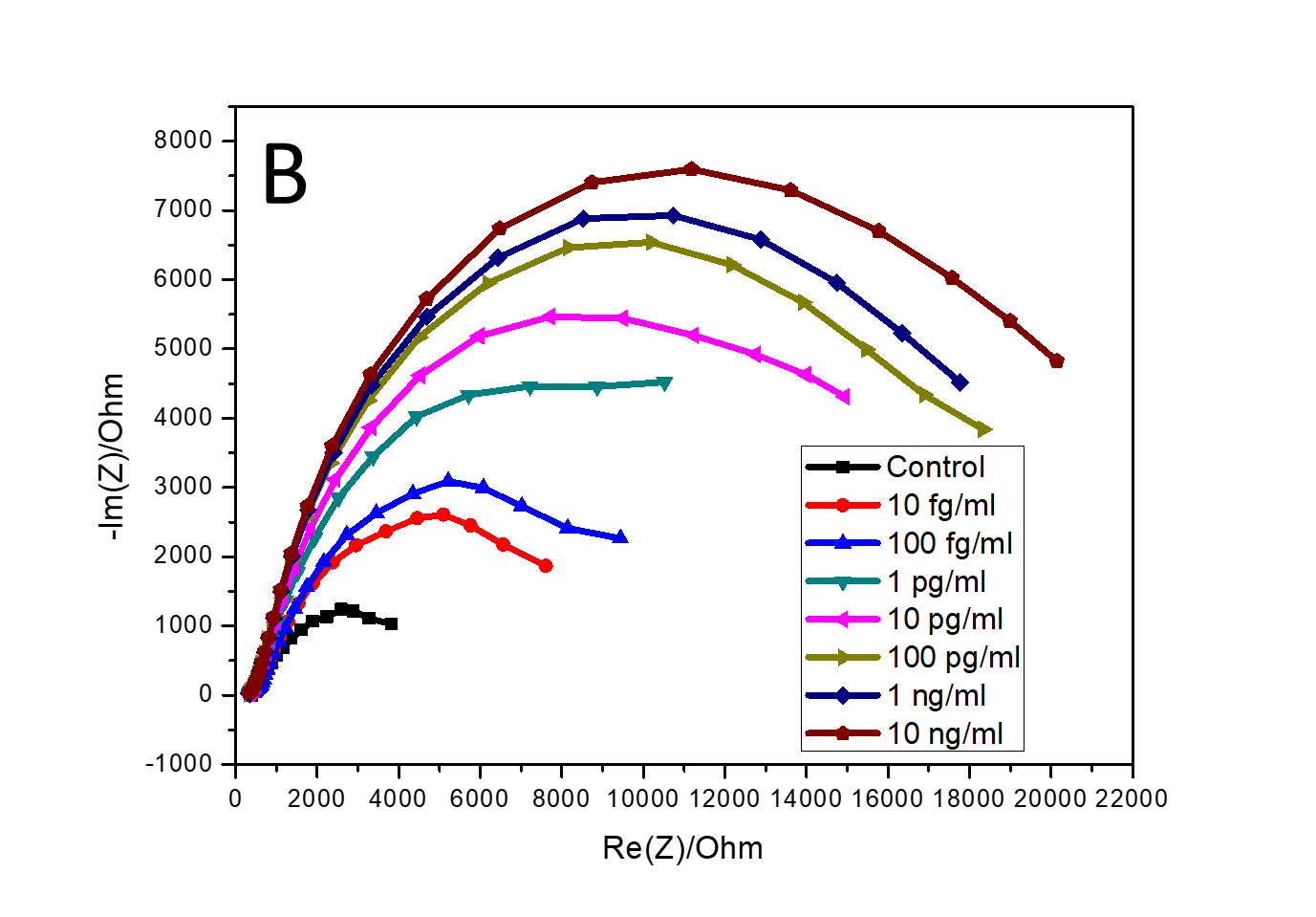


**Fig. S9** Nyquist impedance plots of the Ab-N,S-GQDs@AuNP-PAni/PAni@CSR sensor electrode before and after loading A) HEV and B) influenza virus.


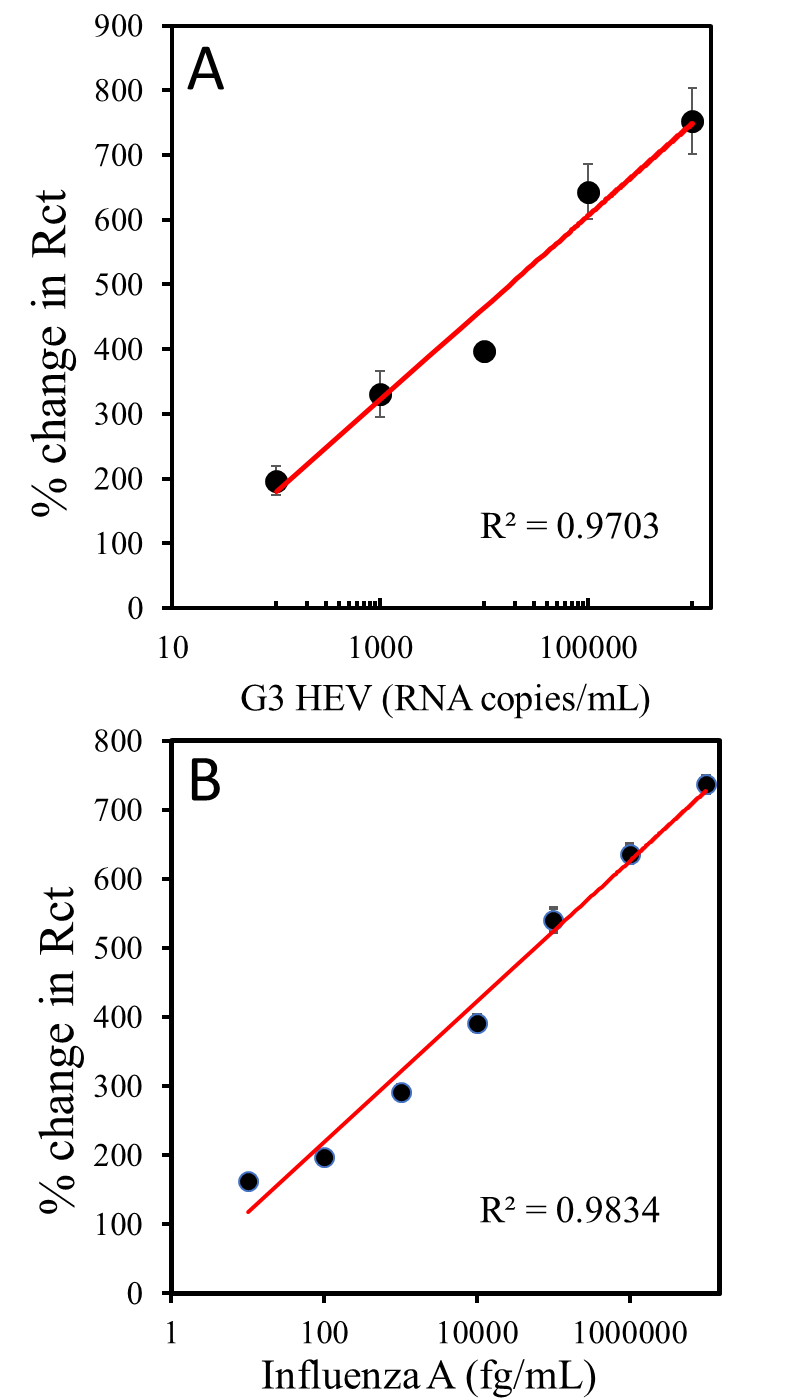


**Fig. S10** Detection result of the genotype 3 HEV (A) and Influenza virus A (H1N1) (B) using the Ab-GQD@AuNP-PAni/CSR sensor electrode, bound with their corresponding antibodies.

1. Correspondence to: Research Institute of Green Science and Technology, Shizuoka University, 836 Ohya Suruga- ku, Shizuoka, 422-8529, Japan. Tel. & fax: +81 54 238 4887. *E-mail address:* park.enoch@shizuoka.ac.jp (EYP) [↑](#footnote-ref-1)
